# Ritualistic Institution and Livelihood Fragility of Female Migrant Workers in Urban China

**DOI:** 10.3390/ijerph17249556

**Published:** 2020-12-21

**Authors:** Chao Wang, Jiayi Tang

**Affiliations:** School of Public Policy & Management (School of Emergency Management), China University of Mining and Technology, Xuzhou 221116, China; 5852@cumt.edu.cn

**Keywords:** female migrant workers, livelihood fragility, family separation, ritualistic institution

## Abstract

China’s rapid urbanization can be attributed, in part, to the contribution of female migrant workers. However, they are a socially vulnerable group. In order to explore the vulnerability of female migrant workers and its reasons, questionnaires and in-depth interviews were conducted with female migrant workers in Guangdong and Hubei provinces, China, and 992 questionnaires and 147 interview data were finally collected as the research object. The descriptive statistical analysis was conducted with the quantitative data to reveal the livelihood vulnerability of female migrant workers and its reasons, and qualitative data were used to corroborate and consolidate the argument. “Ritualistic institution” is the key to understanding the livelihood fragility of female migrant workers. The policy on migrant workers has weakened the concept of family, making it difficult for families, which are on the fringes of the national policy vision, to benefit from the system. Therefore, the livelihood costs of female migrant workers have increased. Traditional gender norms also make it difficult for migrant women to enjoy the limited benefits and resources of the policy. This weakens the authoritative role of the policy in solving the problem of livelihood vulnerability for migrant workers, particularly women. This shows that China’s policy on migrant workers is somewhat symbolic. Through “family separation” and “ritualistic institution”, it can be seen that China’s urbanization is a modern development activity that carries urban bias and lacks humanistic care value. This is bound to result in the neglect of human development, gender differences, and family, making it difficult for rural migrant women to survive. This in-depth study seeks to find solutions to the problems prevalent under the cover of contemporary Chinese modernity.

## 1. Introduction

**Figure 1:**
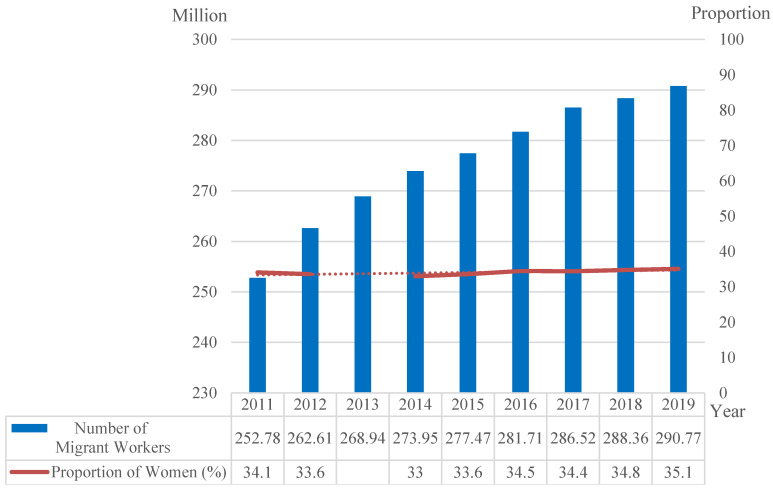
Number and gender distribution of migrant workers in China from 2011 to 2019 (Data source: National Bureau of Statistics. Note: Since the ratio of male to female migrant workers in 2013 is not released by the National Bureau of Statistics, the trend line is used instead here).

With the rapid urbanization in China and the relaxation of the ban on migration between rural and urban areas [1], a large number of rural migrant workers have moved to prosperous urban areas to seek employment opportunities for their livelihood. China saw large-scale migration to cities in the late 1980s and early 1990s. In 1995, the number of migrant workers reached 50 million. After that, the number of migrant workers increased rapidly by 6 million to 8 million every year. In 2019, the total population of migrant workers reached 290.77 million, an increase of 0.8% or 2.41 million from 2018. Women accounted for 35.1% of all migrant workers (Figure 1) [2], and the proportion of women increased by 0.3% from 2018. This not only means that female migrant workers have contributed to China’s urbanization, but also indicates that their livelihood status affects urbanization, because no matter what kind of urban development model is adopted, its execution depends on people. Therefore, the Chinese government has adopted a series of policies for migrant workers to ensure their livelihood, so as to better promote the process of urbanization.

Since 1978, the number of government-issued papers related to migrant worker policy has been increasing in tandem with the rise in the number of migrant workers. The top three years in terms of the number of published papers were 2006, 2016, and 2017. Taking 2002 as the node, the number of policies averaged to about six per year from 1978 to 2002, while the average number rose 9.3 times to about 56 per year from 2003 to 2017. This represents two different stages of development, showing a long-term gradual balance and an occasional transition path. However, the policy on migrant workers based on urban-rural division, urban protectionism, and traditional gender norms poses a series of livelihood vulnerability problems to female migrant workers in the process of creating a strong China with rapid economic development and continuous improvement in overall national strength. The most significant achievement of China’s reform and opening up is the rapid economic development [3]. According to the National Bureau of Statistics, Chinese GDP has grown from 0.325 trillion yuan in 1977 to 99.0865 trillion yuan in 2019, and its per capita GDP increased from 344 yuan in 1977 to 70,892 yuan in 2019. It is difficult for migrant women to enjoy their due welfare, exercise their basic rights, and be accepted by urban society. Thus, they have become socially vulnerable groups.

Migrant workers usually work in the so-called “3D” positions, namely “dirty, dangerous, and demeaning” positions. Compared with male migrant workers, female migrant workers are more likely to be marginalized from urban life [4]. This is due to the fact that the female identity of migrant workers based on gender discrimination, urban-rural dual structure, and urban protectionism makes them relatively more vulnerable to social risks, such as low pay, unemployment, and economic fluctuations [5], facing the risk of social marginalization in employment and life. In addition, they also need to bear the heavy responsibility of childbirth and family labor, which makes them an extremely vulnerable group [6], facing more livelihood vulnerability than male migrant workers [7].

Authors put forward the idea of “ritualistic institution” to interpret the livelihood vulnerability of female migrant workers. When informal rules are superior to formal rules, or the former erodes or even squeezes the function of the latter, the formalization of formal rules will arise. Thus, the system becomes a ritualistic institution, a kind of “symbolic ornament” [8,9]. In other words, the current series of policies for migrant workers is not an appropriate way to solve the problem. The problem lies in the fact that the policy initiatives largely remain on paper. This “ritualistic institution” merely makes a symbolic gesture to the intended audience, covering a chaotic world like a curtain, including various informal behaviors of actors regulated by formal institutions. In fact, the rules of a country or region cannot be comprehended simply by inspecting them superficially and then drawing profound conclusions. Although many institutional rules have been established, some of them are deceptive and could be just symbolic gestures to satisfy the public. It is only when someone does something through the backdoor, that the challenges faced by the victims of such situations in using the formal system provided by the state are revealed [8]. This paper focuses on a group of female migrant workers in Guangdong province and Hubei province to reveal the hollowness of the migrant workers policy and to explore the phenomenon of livelihood vulnerability of female migrant workers.

## 2. Theoretical Framework

The current mainstream view defines “livelihood” as a way of making a living based on capabilities, assets, and activities [10], which has a richer and broader connotation than the concepts of “survival” and “development”, which describe the survival and development of special groups. “Vulnerability” is a widely used concept and refers to the inability of individuals or groups to mitigate, cope with, or recover from disasters. Thus, the concept of “livelihood vulnerability” can be defined as the possibility of sudden risk or damage to an individual or family, or the possibility of the quality of life falling below the normal social living standard.

**Figure 2:**
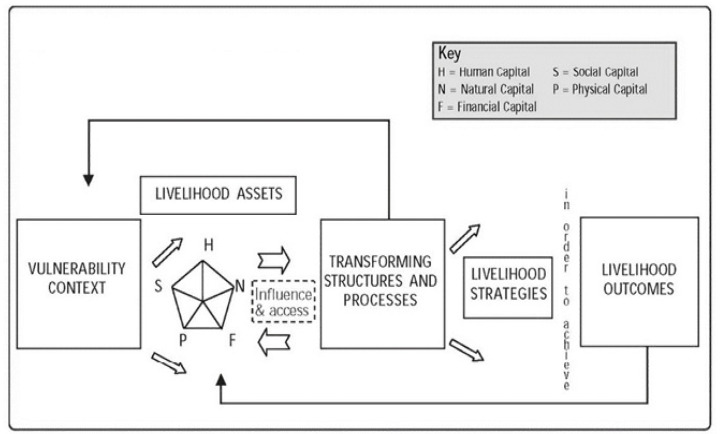
Sustainable livelihood framework established by the Department for International Development [11].

At present, there are many types of interpreting frameworks for livelihood vulnerability, and there is no consensus on these types. Among them, the framework from the UK’s Department for International Development (DFID) is the most prominent and widely used. The DFID analysis framework (Figure 2) outlines how vulnerable individuals pursue different livelihood strategies using a livelihood capital portfolio under the background of structures and processes, thus producing different livelihood outcomes and forming feedback impact on livelihood capital. Due to this, the DFID framework is conducive to the systematic analysis of livelihood vulnerability in the unified logical framework linking macro and micro factors [9]. In the DFID framework, the livelihood system, as the external environment of sustainable livelihood, runs through the whole process of the accumulation and combination of different forms of livelihood capital, the choice of livelihood strategies, the exertion of livelihood abilities, and the outcome of livelihood pursuits. It has a subtle and comprehensive impact on sustainable livelihoods. The importance of institution is emphasized because it is the origin of public policy and has a decisive influence on the whole life cycle of public policy, which leads to different policy outputs from different institutions. Therefore, it is crucial to analyze the vulnerability of livelihood from the perspective of institutions. In this way, the institutional source of livelihood vulnerability can be identified.

North (1990) believes that “system is a social game rule” [12], including a series of formal rules, such as contracts designed according to certain purposes and procedures, and also informal rules such as value beliefs, ethical norms, customs, and habits that people unconsciously form and pass down from generation to generation. Institutional dichotomy has been widely used and studied by scholars from different disciplines around the world, including anthropologists, economists, sociologists, and political scholars [13,14], especially in Latin America and other developing regions [15], Eastern Europe [16], Africa [17], and Asia [18]. There is an interactive relationship between formal rules and informal rules. Formal rules affect the operation track of informal rules, and informal rules strongly affect the function of formal rules. They interact in a kind of complementary and alternative relationship. When they are closely combined, they form a kind of complementary relationship, and when they are separated, they form a kind of alternative relationship. Helmke and Levitsky (2003) divided the relationship between formal rules and informal rules into four types based on their respective features: complementary, adaptive, competitive, and alternative [19].

There have been studies on formal rules and informal rules and their relationships from different perspectives. However, previous studies lack a deep understanding of the impact of formal rules on informal resolution strategies of social actors [12,20]. Institution is a result of rational individuals’ mutual understanding of preference and choice behavior. Failure to comply with these systems is punishable. Therefore, a behavior mode showing a stable state is institution [21]. “Ritualistic institution” conceals the disordered state of anarchy and a world without formal rules. This kind of system only plays an ornamental role, and lacks substantive function. It is easy to prove that such rules are ritualistic. In particular, studies of the actual behavior of actors in the institutional context show that formal rules are not responsive to actual behavior. Of course, the purpose of this study is to go beyond the traditional study of institution and focus on “ritualistic institution”, rather than explore the constituent elements of systems and their relationships.

**Figure 3:**
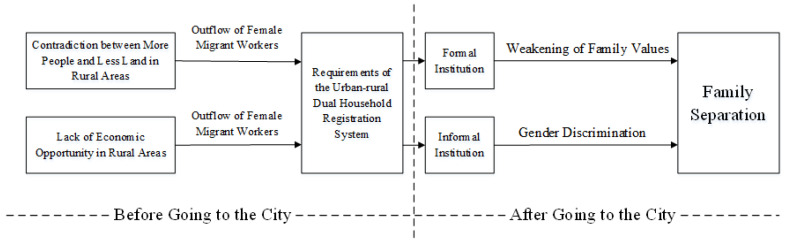
Research framework of this article (made by authors).

This study attempts to reveal the rituality of China’s migrant workers’ policy and explore the livelihood vulnerability of female migrant workers (Figure 3). The contradiction between more people and less land in rural China and the low economic benefits of traditional agricultural production have caused a large number of rural women to migrate to cities in pursuit of higher benefits. However, the current practice of rapid urbanization in China is essentially a developmental thought process which pursues modernity. The most typical example of this is the adoption of the urban-rural dual household registration system to screen out individualized, non-family-led rural elites to enter the city for modernization construction, and refuse to grant them citizenship to share urban welfare resources. Consequently, the migrant worker policies formulated by the city government respond to and accept the efficiency priority rule, adopt the concept of management instead of service to respond to the demands for public interests of female migrant workers, and pay too much attention to the material economy while neglecting the value of care for the family. The policy of migrant workers is an authoritative distribution of social welfare resources. The deep-rooted traditional gender concepts in China often make the distribution of limited migrant workers’ welfare resources gender-biased, making it more difficult for female migrant workers to enjoy policy benefits than male migrant workers, which not only inhibits the authoritative role of migrant workers’ policy in solving the livelihood vulnerability of female migrant workers, but also exacerbates the livelihood vulnerability of female migrant workers.

## 3. Data Sources and Research Methods

### 3.1. Ethics Statement

Well-trained and professional investigators carried out face-to-face questionnaire surveys and semi-structured interviews. Investigators were required to give a brief introduction regarding the research and the questionnaire to every respondent; the respondent was then asked to fill out the questionnaire independently. It took about 15 to 20 min to complete the questionnaire and about 30 to 40 min for each semi-structured interview, and all respondents were compensated for their time. The questionnaire and in-depth interviews were confidential and anonymous. Written informed consent was obtained from all respondents to indicate that participation in this research was completely voluntary. In addition, we optimized and refined the expressions of respondents without changing their original intention to improve the readability and comprehensibility of the article.

### 3.2. Selection of Research Sites

This research used a questionnaire and in-depth interviews to collect data, and chose Guangdong province and Hubei province in China as the research site. Due to differences in economic and social development levels, the central and eastern regions have quite different attractions for migrant workers. The migration type of migrant workers in the eastern region is mainly inter-provincial migration, while the migration type of migrant workers in the central region is mainly inter-city migration within the province [22]. Guangdong province and Hubei province are located in the eastern and central regions of China respectively, and both are densely populated areas where migrant workers are concentrated. Therefore, to a certain extent, these two provinces are of representative significance for studying the issues of female migrant workers. Based on this, the research took Guangdong and Hubei as research areas. In the selection of specific research sites, the differences in urban class, scale, and urbanization degree between different cities were considered mainly [23]. Finally, seven cities in Guangdong and Hubei were selected as specific survey sites, including two large cities, two medium cities, and three small cities.

### 3.3. Process of Field Investigation

The questionnaire survey adopted multi-stage, stratified, and random sampling methods to select survey samples. First, the survey districts were selected based on the characteristics of the cities, with the survey scope covering all municipal districts for the large city, and one or two districts for the medium-sized and small cities. After that, the communities and enterprises where the female migrant workers were concentrated were selected as specific research sites to ensure the rational distribution of research forces and the scientific distribution of research sites. According to the principle of “basic balance of occupation and age”, with female migrant workers as the sampling unit, and simple random sampling or systematic sampling for probability sampling. A total of 1100 questionnaires were distributed, and 992 valid questionnaires were returned, with the effective rate being 90.2%. Among all valid questionnaires, 454 were from Guangdong province and 538 were from Hubei province. The questionnaire mainly focused on the human capital, social capital, financial capital, and physical capital of female migrant workers, and consisted of seven parts: basic life and employment, marriage and family, occupational safety and social security, social relationships, mental health, self-identity and development, and background information.

The in-depth interview was to obtain a deeper understanding of the vulnerability of female migrant workers and the interviewed female migrant workers were selected through purposeful sampling rather than random probability sampling. A “snowball” method was adopted to fully understand the livelihood of female migrant workers. As most female migrant workers were employed in the service, manufacturing, textile, housekeeping, and wholesale industries, 133 female migrant workers working in these industries were initially invited to participate in interviews in Guangdong and Hubei. In order to test whether these cases were sufficient to represent the diverse livelihoods of female migrant workers, 14 additional interviews were conducted, and no novel cases were found. Therefore, this survey finally conducted 147 interviews in Guangdong and Hubei. The in-depth interview was conducted in the form of the semi-structured interview, and questions of the interview, like the questionnaire, also focused on the human capital, social capital, financial capital, and physical capital of female migrant workers. The data obtained from interviews were used as cases to corroborate and consolidate our argument.

## 4. Major Findings

### 4.1. Most Female Migrant Workers Are Rural Elites

Most of the rural female laborers working in cities were in their 20s, 30s, and 40s. Compared with rural left-behind groups (the elderly, children, and other people who lived in rural areas and had difficulty adapting to urban work and life), they had advantages in health conditions, energy, and educational attainment levels, which made them rural elites. Because of this, they could move out of rural areas, and get higher economic benefits in cities.

First, most female migrant workers were new generation women. In order to understand the age distribution of female migrant workers, we divided the age of the questionnaire respondents into three stages, and set up three questionnaire options: born before 1980, born 1980–1990, and born after 1990. According to the data from the questionnaire survey, the age structure of female migrant workers was dominated by the new generation (born after 1980), and the new generation of female migrant workers accounted for 60.4% of total female respondents, of which 39.6% were born between 1980–1990 and 20.8% were born after 1990 (Table 1). The possible explanation for the characteristics of the age structure of the survey samples was that the post-1980s and post-1990s had a lighter rural complex and less dependence on land than for those women born before 1980. Meanwhile, they were better able to adapt to the development of society and accepted new things faster. They were eager to broaden their horizons and enrich their life experiences in the bustling city. Therefore, the new generation of female migrant workers had increasingly become the backbone of urban construction. However, for female migrant workers who were born before 1980, the responsibilities of taking care of their families and the desire to settle in their hometowns had caused them to end the “migratory bird-like” life in cities as they grew older. Therefore, the proportion of female migrant workers born before 1980 was relatively low.

**Figure 4:**
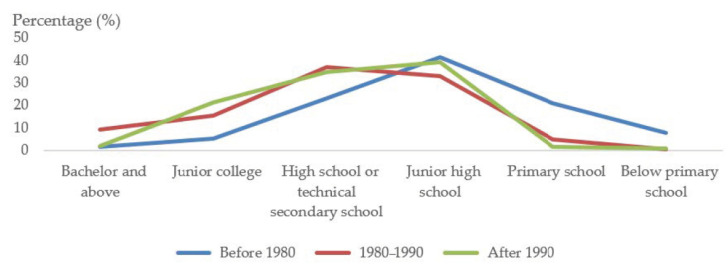
Educational levels of samples at different age levels.

Second, most female migrant workers had a higher education level than those in rural areas who did not work in cities. The survey data showed that 48.5% of female respondents had received high school education or above, and only 3.2% of them had an education level below primary school (Table 1). Among the female migrant workers interviewed, 43.3% of those who worked in the provincial capital had received high school education or above, while those in counties had mostly completed the junior high school education level. The data reflect the characteristics of rural women who migrate to cities and towns. Generally, women who choose to leave rural areas tend to have a certain level of education. Most of the women with a high educational attainment level migrate to big cities, while those with relatively low education levels relocate to small towns. In addition, the educational level of female migrant workers varied greatly among different age groups. Among the female migrant workers who were born before 1980, 1980–1990, and born after 1990, the proportions of those with a junior college degree or above were 6.6%, 24.4%, and 23.3%, respectively, showing a trend of growth with generational change. What was particularly gratifying was that the proportion of the sample with a primary school education and below was 28.8%, 5.3%, and 2.4% in the above three age groups respectively, showing a trend of a significant decline with the generational change (Figure 4).

The reason why most female migrant workers are rural elites needs to be analyzed in the context of urban and rural social reforms in China. First, the reform of the rural household contract responsibility system transformed the rural landscape, resulting in a surplus rural labor force. The reform liberated a considerable proportion of agricultural laborers. At the same time, it also reduced the absorption capacity of the agricultural labor force, as a result of which labor supply exceeded demand in the period of planned economy. Moreover, with the popularization of new agricultural technologies, the demand for manual labor in agricultural production started decreasing. The limited arable land resources in rural areas further reduced the demand for labor. Thus, the problem of rural labor surplus became increasingly pronounced. Before the mid-1980s, the proportion of rural labor surplus in China was 30–40%, and the surplus in 1986 was 114–152 million [24]. In the 1980s, the proportion of rural labor surplus in China was more than 30% [25]. Compared with the 1980s, the number and proportion of China’s rural surplus labor force showed an upward trend in the 1990s. Since the beginning of the 21st century, however, the growth in the number and proportion of rural surplus labor force has slowed down. In 2001, there were 482 million rural laborers in China, which increased to 498 million in 2002. Since then, the average annual growth rate has been 10 million [25]. In other words, a large proportion of the rural surplus labor force needed to be transferred from agriculture.

**Figure 5:**
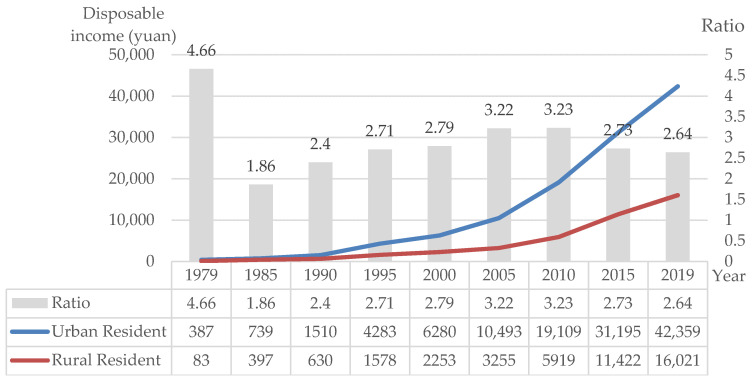
The annual disposable income of urban and rural residents from 1979 to 2019 in China (Data source: National Bureau of Statistics).

Second, because of the lack of employment and economic opportunities, a large number of young and middle-aged rural women have poured into cities and towns from rural areas in pursuit of a better livelihood. After the founding of new China, the state implemented the strategy of prioritizing the development of heavy industries, accumulating funds for industrial development through scissors-gap prices for industrial and agricultural products. After 1979, the rural labor force, which had been bound to the land for a long time, could flow to the city. The industry extracted cheap labor resources from the countryside, which helped industrialization progress at a fast pace. The long-term parasitic state of industrialization and agricultural modernization made it difficult for rural surplus labor to transfer to the secondary and tertiary industries. From the perspective of an industrial economy, the economic added value of agriculture is far lower than that of the manufacturing and service industries. It is difficult for farmers to obtain higher economic income by relying on traditional agricultural production. Since 1978, the income gap between urban and rural areas in China has generally been widening. In 2015, the per capita disposable income of urban residents was 31,195 yuan, while the per capita disposable income of rural residents was 11,422 yuan, meaning the income of urban residents was 2.73 times that of rural residents (Figure 5). In order to seek better development opportunities and higher economic benefits, it became inevitable for rural surplus labor to move to cities, and Ms. Zhao was one of them.

Ms. Zhao found a job as an urban sanitation worker under the introduction of her fellow villager. When she talked to us about her job, she said: “I have been in this job for 5 years. It is good, and I don’t think it is hard or tired because I was used to doing farm work before. And the good news is that I can earn 4000 yuan a month now, while I can only earn 1000 yuan a year in rural areas! I have some savings now, and I plan to buy a small house in the city in a few years!”

(personal communication)

**Figure 6:**
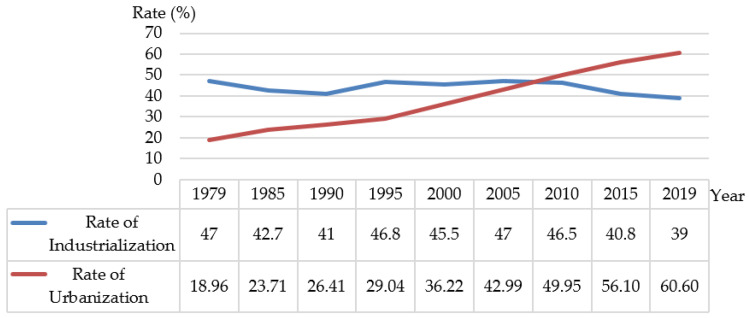
Rate of industrialization and rate of urbanization from 1979 to 2019 in China (Data source: National Bureau of Statistics).

Third, the urban-rural dual household registration system required a suitable rural labor force for modernization. Naturally, the rural labor force flowing into the city was the elite of the countryside. After the reform and opening up of the economy, the rural surplus labor force transferred to cities and towns on a large scale, and the urbanization speed also accelerated [26]. Urbanization continued to grow from 18.96% in 1979 to 60.60% in 2019, but there was a gap compared with the floating industrialization rate of about 45% (Figure 6). Urbanization lagged behind the industrialization process for a long time, as a result of which cities could not provide rural migrant workers with the infrastructure and basic public services needed for survival and development. To ensure the smooth progress of modernization with industrialization as the core, the Chinese government implemented a matching household registration system. A set of urban bias policies with household registration as the core was developed to prevent the large-scale flow of rural labor force and ensure the supply of urban basic living goods and minimum social welfare as the starting point [27]. This kind of urban-biased household registration system arrangement naturally requires the rural labor force that enters the city for employment to be the rural elite. They have high human capital and can cope with the problem of insufficient supply of welfare resources and public services with their own human capital.

### 4.2. Female Migrant Workers Face the Problem of Livelihood Vulnerability

There is no consensus on the connotation of the concept of livelihood, but it is generally recognized that livelihood capital is the core component of livelihood [28]. Moser (1998) believes that the ability to achieve different livelihood strategies depends on the social and material capital owned by individuals or families [29]. He divided livelihood capital into four categories: human capital, social capital, natural capital, and financial capital. The DFID framework, established in 2000, divides financial assets into physical capital and financial capital. Existing research also divides the livelihood capital of migrant workers into human capital, social capital, financial capital, and material capital [30]. Based on these classifications, this study evaluated the livelihood vulnerability of female migrant workers from the perspective of livelihood capital. The economic value created by land resources is low, especially when migrant workers relocate to cities, so natural capital was not considered in this research.

#### 4.2.1. Financial Capital Is Virtual 

Financial capital is the accumulation and flow of economic resources that people need to achieve their livelihood goals [31], with money directly related to their livelihood status and their ability to prevent and repair livelihood vulnerability. On the basis of the questionnaire survey, the average monthly income of female migrant workers was less than 2300 yuan (Note: The first maximum monthly income of questionnaire respondents was 30,000 yuan, the second maximum was 10,000 yuan, the third maximum was 8000 yuan, the fourth maximum was 7000 yuan, and the fifth maximum was 6000 yuan. The sample size of each maximum income did not exceed 5, and the female migrant workers involved were self-employed. These samples had widened the income gap of female migrant workers, so it would be more representative to exclude these 13 samples. Therefore, a total of 979 samples have been calculated for average monthly income). According to the National Bureau of Statistics, the average monthly income of migrant workers in 2015 was 3072 yuan, which means the average monthly wage of women interviewed was much lower than the national average. When it comes to the daily consumption expenditure of female migrant workers, 62.4% of their wage income was used for daily meals and 21.6% for housing expenses. In addition to daily consumption, 41.2% of the surveyed women spent their income on savings and 30.6% chose to use it on their children’s education. The study found that 10.0% of the women had no money left after spending on daily consumption items (Table 2). By analyzing the structure of income and expenditure, it was found that children’s education expenditure undoubtedly increases the economic burden of female migrant workers. The situation of Ms. Wang reflects the heavy economic pressure faced by migrant women working in the city.

Ms. Wang, a single mother with a three-year-old daughter, can only hire a nanny to look after the child because she is working alone in the city with her child. However, the cost of hiring a nanny is very high; she has to work two jobs to make ends meet. Ms. Wang said that when her daughter is older, she will go to kindergarten. The child can only go to private kindergartens because she does not have a registered permanent residence in the city. The tuition fees of private kindergartens are very expensive; therefore, Ms. Wang intends to take her daughter back to her hometown and ask the child’s grandmother to take care of the child until she earns enough money for tuition fees.

(personal communication)

#### 4.2.2. Human Capital Is Insufficient

In the era of the knowledge economy, education level and vocational training in the later stages are related to the sustainable livelihood ability and livelihood recovery ability of individuals. However, the vocational training scenario for female migrant workers is not optimistic. According to the survey data, only 23.6% of questionnaire respondents had a professional qualification or technical level certificate (Table 2). Due to the lack of vocational and technical education and training, most female migrant workers are forced to stay in the informal sector of the low-end secondary labor market and engage in “3D” (dirty, demanding, dangerous) jobs that urban residents do not want [32], which affects their career development path in cities and towns. According to the survey, 55.7% of the surveyed women worked in labor-intensive industries with low requirements for professional skills and quality, while only 35.7% were engaged in management, professional, and technical occupations, indicating that most of the female migrant workers were at the bottom of the occupational pyramid. Ms. Yi is a typical female migrant worker suffering from a lack of human capital.

Ms. Yi followed her husband to work in the city after getting married. Due to her low educational level and lack of professional skills, she was engaged in cleaning work at a hotel. Her exposure to hydrochloric acid in toilet cleaners for a long time every day often made her feel dizzy and sick. She later found out that she was pregnant, but the doctor told her that harmful chemicals had caused fetal dysplasia, forcing Ms. Yi to terminate the pregnancy, which almost broke her.

(personal communication)

#### 4.2.3. Material Capital Is Weak

Material capital refers to the infrastructure, mainly housing, that migrant workers need to maintain their basic livelihood [33]. Housing is a basic necessity, as migrant workers need a place in cities and towns where they can settle down and live. However, 39.0% of the surveyed female migrant workers said their housing needs were not met, forcing family members to live apart in separate spaces, instead of living together in the same place. Of the surveyed female migrant workers, 68.3% were not satisfied with their living conditions (Table 2). There were two reasons. First, there were rampant theft, fighting, gambling, unlicensed business, unlicensed medical practice, and other illegal activities in their living environment, which increased their fear and mental stress. Second, poor living conditions and the resulting changes in living habits caused them to suffer from obesity [34], rheumatism, respiratory diseases, and gynecological diseases, which not only increased their physiological and economic burden, but also affected their work efficiency and economic income. The purpose of migration for female migrant workers is to pursue a better life, but their dissatisfaction with the living conditions reduces their happiness with urban life. Ms. Zhang has been working in the city for nine years, but she does not have even normal “housing.”

Ms. Zhang, a divorcee, works alone in the city, with her two children staying at her rural home. Due to the heavy economic burden of taking care of her family, she has no money to rent a house in the city and lives under a canvas canopy on undeveloped land. The “housing” is very dangerous, and it is inconvenient for her to bathe or use the toilet.

(personal communication)

#### 4.2.4. Social Capital Is in Shortage 

Social capital, which is part of social network relations, social organizations, and social systems, is a resource that people can use in the process of pursuing their livelihood [35]. China’s household registration system is not only a basic population management system, but also a social shielding system embedded with many resources of interest. It prevents rural migrant workers from sharing urban resources, thus constantly reinforcing the differences in social relations and strengthening the class superiority consciousness of urban residents. Urban residents tend to view vulnerable rural migrant workers as people that are inferior, disobey rules, destroy the order of the city, and compete for the scarce public services and resources in the city [36], and this view is reflected in their attitude toward female migrant workers. According to the survey data, 31.3% of the survey women thought that city residents were arrogant and looked down on country people (Table 2). This prejudice increases the sense of alienation between the migrants and urban residents. As a result, the communication between the two groups generally revolves only around business relations. This undoubtedly aggravates the “involution” of social communication and leads to isolated communication between “two types of residents dwelling in one city.” The survey data showed that 17.9% of questionnaire respondents said that half of the friends they made in the city were migrants, 32% said that most of the friends they made in the city were migrants, and even 8.5% of the women interviewed said that all friends that they made in the city were migrants (Table 3). It can be seen that a considerable number of women migrant workers have not included urban residents in their social circle. It is undeniable that there are interactions and associations among female migrant workers in cities, however, they are all from rural areas, and have similar living environment and social capital, this homogenous association is difficult for them to expand the social network and increase the social capital. 

### 4.3. Ritualization of Policy for Migrant Workers: An Important Factor Influencing the Livelihood Vulnerability of Female Migrant Workers

#### 4.3.1. The Weakening of Family Values

The policy on migrant workers has weakened family values and raised the living costs of female migrant workers. China’s constitution has always focused on the spirit and essence of family. However, China’s modernization process in the past 100 years has consistently undermined family values and sacrificed family interests [37]. Consequently, the basic unit of modern society has sunk to the individual level. Family is on the fringes of the national vision, and, therefore, finds it difficult to be the beneficiary of state policy. The weakening of the family concept in the migrant workers’ policy has impaired the normal functioning of the family structure of migrant workers, raising the living costs of migrant women in urban society.

First, the urban housing security policy ignores family needs. Article 20 of the “Administration Measures of Affordable Housing in Wuhan City, Hubei Province” (2009) stipulates that only when “a family member has a permanent residence in the city” can a person apply for the purchase of affordable housing. However, rural migrant workers do not have urban residence registration, so it is difficult for them to apply for affordable housing. In May 2013, Wuhan issued the public rental housing policy, which included the floating population from outside the city within the scope of public rental housing for the first time. It generally requires an applicant to have a college degree or above, sign a fixed labor (employment) contract with the employer for one year or more, and pay social insurance or contribute to the housing accumulation fund. However, according to the survey data, only 15.4% of the female respondents had an education level of junior college or above (Table 1), 35.2% had not signed labor contracts with employers, and 87.5% had no provident fund (Table 2). In fact, most of the women were not eligible to apply. In addition, there are many problems in affordable housing and public rental housing for migrant workers. “The Opinions of Hubei Provincial Government on Further Strengthening the Housing Security Work” (2010) stipulated that “in cities, development zones and industrial parks where migrant workers are relatively concentrated, affordable rental housing conforming to the characteristics of migrant workers and meeting the residential needs of migrant workers shall be intensively constructed.” However, this kind of resettlement area is mostly located in the suburbs far away from the city, which objectively separates migrant workers from urban society.

Second, the employment support policy is inadequate. Survey data showed that 45.8% of migrant women in cities had to go back to the countryside because they needed to take care of the elderly and children. The most practical and effective way to help female migrant workers achieve a balance between work and family responsibilities is by solving the problem of supporting the elderly and addressing childcare issues. However, the current child care policies, such as child care subsidies and free child care policies, as well as elderly or patient care policies, which are targeted at rural migrant families, are still in a state of deficiency and cannot reduce the burden of family care for female migrant workers. For example, the individual tax system does not take into account the overall situation of tax-paying families and the cost incurred by rural migrant workers on providing care for the disabled elderly or patients. At the same time, urban employment policies lack rigid regulations and disciplinary rules that require employers to take on the responsibility of helping women balance family and work, including by providing elderly care and patient care services. Migrant workers find it difficult to pay for private care due to income constraints. Female migrant workers have to juggle family and work, give up full-time work and engage in part-time jobs, choose intermittent employment, or even quit their jobs. This is bound to negatively affect the income level of migrant families. When it becomes difficult to maintain the high cost of living in cities and towns, they are forced to return to the countryside.

Third, the social security policy lacks provisions on family care. In addition to the minimum living security system for urban families, the existing social security policies are all aimed at individual citizens. The minimum living standard security system for urban residents can provide certain guarantees for the basic life of poor family members. However, in Hubei province, those who apply for minimum living security must also have local residence or non-agricultural household registration. The restriction of urban household registration makes it difficult for rural migrant families to enjoy subsidies. As much as 94.1% of the female migrant workers interviewed did not enjoy the urban minimum security system. This would result in a lack of ability to resist risks. If an accident occurs, the family economy will collapse and make it difficult to maintain their family life in cities and towns. At the same time, the survey data showed that the participation rate of the women interviewed in medical insurance, endowment insurance, work-related injury insurance, unemployment insurance, maternity insurance, and the housing accumulation fund was not satisfactory, with the proportions at 48.3%, 33.9%, 25.3%, 18.8%, 14.0%, and 12.5%, respectively (Table 2). This means that they face greater risks in terms of unemployment, illness, and work-related injuries. Ms. Chen was one of these migrant women.

Ms. Chen worked in a domestic company, which provided her with neither medical insurance nor endowment insurance. None of the seven companies she had worked for before provided her with insurance. She said that infusions and medicines were expensive in urban hospitals, so she was most afraid of getting sick when working in cities.

(personal communication)

Fourth, the allocation of public education resources is localized. On 8 January 2013, Hubei province issued the “Opinions on Securing Opportunities of Entrance Examination for Migrant Workers’ Children after Receiving Compulsory Education.” However, this document requires the father (mother) or legal guardian of the accompanying children to have a legal and stable occupation and have a legal and stable residence (including lease). This ignores the high mobility and dispersion of rural migrant workers. According to the survey data, 36.6% of the respondents had changed their work units and 75.8% had changed their residences in 3 years (Table 2). Due to the localization tendency of urban basic public education resource allocation, migrant workers’ families bear the heavy burden of their children’s education, which forces them to leave their children behind in rural areas to study, even as they worry about their children while working in cities and towns. Despite the implementation of the policy of free compulsory education in public primary and secondary schools, local governments, that are resistant to the idea of “cultivating other people’s children with their own money”, have set up artificial barriers for migrant workers’ children to enter school. Public schools offer limited degrees to migrant children, forcing the vast majority of migrant workers’ children to attend private schools with high fees. These factors have increased the economic burden of migrant workers’ families, while dampening the enthusiasm of their children in pursuing education.

#### 4.3.2. Gender Stereotyping

Traditional gender norms make it difficult for migrant women to enjoy the benefits of migrant worker policies in an equitable manner, inhibiting the authoritative role of such policies in solving problems of livelihood vulnerability. Traditional gender norms shape the gender roles of rural women and family expectations. Traditional virtues such as “carrying on the family line”, “assisting the husband”, “bringing up children”, and “realization of husband’s achievements” are regarded as the standard to measure the value of a woman’s life [38]. This limits women’s activities to household chores. Although the policy on migrant workers lacks family value, the traditional gender concept hinders migrant women from enjoying the limited benefits and resources that are part of the policy. This makes migrant women vulnerable to risks and hinders the implementation of policies for migrant workers. In other words, there is an informal “underground world” that ignores the power and authority of the formal system [8].

First, female migrant workers are exposed to limited vocational skills training. They are forced to stay at the bottom of the occupational pyramid, and their income is generally lower than that of men. The “spillover effect” of human resources can make other people, enterprises, society, and even the country benefit from this resource [39], which is also an important driving force for the state to formulate training policies for migrant workers. However, most of the current vocational skills training policies for migrant workers are gender neutral, which results in the neglect of female migrant workers. Moreover, due to the lack of restraint and supervision in the training system, few employers have placed vocational training of migrant women on their agenda. This hinders women’s career advancement in cities and towns. Of the surveyed women, 33.4% believed that the most important factor hindering their development in cities and towns was the lack of employment skills (Table 2). At the same time, gender discrimination in the field of employment has squeezed the employment space for female migrant workers. The Labor Law of the People’s Republic of China has clearly stipulated that it is illegal to refuse employment or higher employment standards on the grounds of gender. However, it is common for women to be discriminated against and face prejudice in employment because of their gender, which is mainly manifested in industry segregation and occupational restriction. Thus, female migrant workers are forced to work in employment fields that require a low level of skills and have limited prospects for career development, which is the reason why this group has more low-income and less high-income people.

Second, occupational safety protection for female migrant workers is insufficient. Female migrant workers live in an unhealthy working environment, which affects their sustainable development in the city. The “Special Provisions on Labor Protection of Female Workers” (2012) and other policies and regulations require the protection of women’s safety and health in the workplace. However, according to the interview, few female migrant workers have enjoyed special protection during menstruation, pregnancy, childbirth, and lactation. In addition, they face sexual harassment from time to time. Due to the traditional gender culture, even if they are sexually harassed, they usually choose to conceal such incidents, instead of seeking justice in courts, because of the fear of gaining a bad reputation. Even if they win a lawsuit, the publicity of their sexual harassment would be a “scandal” that could even result in the loss of their jobs. Sexual harassment not only makes women “unable to work normally”, but also increases their sense of fear and crisis in urban life. The original labor contract can be used to effectively protect the legitimate rights and interests of rural migrant workers. However, only 64.8% of the women were interviewed for labor contracts (Table 2). This means that their basic legal rights and interests are difficult to protect in the case of labor disputes and occupational injury. This also increases the uncertainty and instability of their lives in cities and towns. The case of Ms. Xiao reflects the unsafe factors faced by female migrant workers when they work.

Ms. Xiao, working in the “entertainment city”, said that she must get used to potential sexual harassment from clients because she was paid based on work achievements. If she did not please her clients, she would not be able to earn money.

(personal communication)

Third, female migrant workers face discrimination and marginalization in politics, which weakens their social capital “reproduction” ability. Political participation enables the formulation and implementation of policies that reflect fairness and justice and promote the equitable distribution of resources [5]. However, due to the influence of the patriarchal political culture on women’s political participation in China, the power and resources to manage state and social public affairs are dominated by men. Even if they enter the political system, women are expected to effectively fulfill their family roles as good wives and mothers. Thus, such women face conflicts between career and family, which may force them to give up their political role and return to their family role. Moreover, the current political participation system lacks gender sensitivity. This is mainly reflected in the fact that the revision of the political participation system lags behind the emergence of gender issues. In 2012, China’s Ministry of Civil Affairs issued the “Opinions on Promoting the Integration of Migrant Workers into Urban Communities”, but the measure did not address the issue of rural migrant workers’ participation in elections in urban areas. As a result of institutional arrangements, 73.2% of the women interviewed had never participated in community democratic management activities, 74.4% had never participated in community democratic elections, and 50.6% had never participated in party and league activities (Table 2). Nowadays, participation in public political life is not only a conspicuous sign of the expansion of political participation, but also an important way to accumulate social capital. Marginalization of women in politics not only deprives the public policy-making process of the voice of female migrant workers, but also weakens women’s ability to reproduce social capital.

## 5. Discussion

From the beginning, the identity of female migrant workers in urban and rural areas, the household registration system, and gender norms have implied that they are a vulnerable group in urban society. The weakening of the idea of family in urban public policy has damaged the structural function of migrant workers’ families. This has not only raised the living costs of female migrant workers, but also increased their likelihood of returning to the countryside because of their family’s difficulties in living in cities and towns. As China takes rapid strides toward urbanization, economic growth dominates everything and constitutes the priority target, which is essentially modern development thinking. The state defines the public-private boundary of the family according to the needs of modernization. The typical method is to use the urban and rural dual household registration system to select rural labor best suited for the modernization activity. This requires migrant workers to be rural elites that have left their families behind and seek no share in urban welfare resources. In such a scenario, it is inevitable that the development process ignores people, gender differences, and family values. The migrant worker policy born amidst urbanization also accepts the rule of prioritizing efficiency. The development of the material economy has been overemphasized, while the fundamental purpose of making people’s lives better has been ignored.

Public policy is a tool for the authoritative distribution of social resources. Traditional gender norms have affected the distribution of interests, thus inhibiting the authoritative role of migrant workers’ policies in solving the livelihood vulnerability of migrant women. Female migrant workers not only encounter the same problems faced by male migrants, but also put up with unique challenges caused by gender differences. The Chinese traditional gender culture confines women’s activities to trivial family affairs and regards men’s achievements as the realization of their own value, which marginalizes female migrant workers in the process of urban resource allocation [9]. Although female migrant workers have made great contributions to urban development and boosted their family income, they are in a state of competition with men for limited urban employment resources. Fierce competition may even reduce the wage level of this group, resulting in conflicts and exclusion within the rural migrant community. Moreover, the long-term urban biased development policy has solidified the distribution pattern of interests between urban and rural areas. A large number of rural women working in cities will inevitably compete for the original limited urban resources, which will lead to the exclusion of female migrant workers in urban society.

China’s policy for migrant workers is symbolic and hollow to a certain extent, revealing how China’s urbanization process has not yet stepped out of the practical dilemma of undertaking modernization while promoting humanistic care values. The process of development has neglected human and family values and ignored the differences between men and women, which raises the risk of livelihood vulnerability for female migrant workers. This study provides a perspective on the very real, social, and practical world hidden behind the formal system, which often has only an ornamental purpose. It reveals the chaotic world of informal behavior of various social actors under formal rules, and reflects the reality of Chinese society. Social actors do not rely on the formal institutional rules in Weber’s (1922) sense [40], but use informal institutional channels to solve social problems, putting those who lack resources and institutional rights at a disadvantage. Therefore, the existence of “ritualistic institution” hinders China’s development into a country ruled by law in the true sense, while the vulnerable groups in society remain in a state that lacks dignity.

From the perspective of the development of human civilization, the path to social justice and the betterment of human life relies on good-law governance, that is, the “rule of law.” To this end, China must clarify the provisions on gender equality from the legal level, formulate policies that specifically take the family as the basic unit, and incorporate sensitivity toward family and gender equality into the mainstream policy agenda for migrant workers in accordance with the law, and consider families at the edge of the country’s vision and female migrant workers at the bottom of society as the beneficiaries of the institution. At the same time, it is necessary to establish a family welfare policy system for migrant workers and a system of supervision and law enforcement for gender equality that are compatible with economic and social development and can also promote the coordinated development of the entire society, so as to protect the family rights of migrant workers and the legitimate interests of female migrant workers from a legal perspective. The goal should be to create a “family-oriented” social and ecological environment that ensures that migrant workers, including female migrant workers, can get equal opportunities to decent and dignified employment and a happy life.

## 6. Conclusions

This empirical study was carried out in Guangdong and Hubei. Through the analysis of questionnaire data, it was found that most of the migrant women flowing into the cities are elites in rural areas, and they play a significant role in the construction and development of Chinese cities and towns. The key to urbanization is the urbanization of people. We need to pay attention to people’s quality of life, development potential, happiness index, and let people enjoy a happier and more dignified life. However, the weakening of the concept of family by migrant workers’ policies has led to the separation of migrant workers’ families, impairing the normal functions of migrant workers’ families, and thus increasing the livelihood costs of female migrant workers in urban society. The traditional gender perspective makes it difficult for female migrant workers to equally enjoy the benefit resources of migrant workers’ policies, and inhibits the authoritative role of migrant workers’ policies in solving the problem of livelihood vulnerability. As a result, in the process of creating a strong China, female migrant workers encounter a host of sustainable livelihood problems. The livelihood of this group faces threats in urban society, which are manifested in the form of lack of human capital, material capital, financial capital, and social capital [41], which may evolve into social instability factors and aggravate disorderliness and risks in society. This gives rise to the question of how China can achieve fair and effective modernization, and puts forward demands and hints for the improvement and optimization of migrant workers’ policies.

## Figures and Tables

**Table 1:** Demographic information of questionnaire respondents.

Variables	Frequency (N = 992)	Percentage (%)
Year of birth		
Before 1980	393	39.6
1980–1990	393	39.6
After 1990	206	20.8
Education level		
Bachelor and above	45	4.5
Junior college	108	10.9
High school or technical secondary school	328	33.1
Junior high school	381	38.4
Primary school	98	9.9
Below primary school	32	3.2
Marital status		
Single	355	35.8
Married	614	61.9
Divorced	18	1.8
Widowed	5	0.5
Current work location		
Capital city	600	60.5
Prefectural-level city	174	17.5
County	212	21.4
Town	6	0.6

**Table 2:** Key variables of female migrant workers’ livelihood vulnerability in China.

Variables	N	Percentage (%)	Variables	N	Percentage (%)
Obtaining a professional qualification or technical level certificate			City residents are arrogant and look down on country people		
Yes	234	23.6	Yes	310	31.3
No	758	76.4	No	682	68.7
Spending on daily consumption items			The main personal factors hindering your development in cities		
Meal	619	62.4	Lower educational background	407	41.0
Clothes	100	10.1	No skill	331	33.4
Housing	214	21.6	Poor adaptability	31	3.1
Phone bill	23	2.3	Gender	9	0.9
Traffic	10	1.0	Poor communication	75	7.6
Medical treatment	6	0.6	Low self-esteem	22	2.2
Others	20	2.0	Others	117	11.8
Highest spending after daily consumption expenses			Housing needs can be met		
Savings	409	41.2	Yes	605	61.0
Education of children	304	30.6	No	387	39.0
Entertainment	97	9.8	Satisfied with the living conditions		
Interpersonal relationships	29	2.9	Yes	314	31.7
Investment	9	0.9	No	678	68.3
Self-learning and improvement	20	2.0	Enjoy the urban minimum security system		
No money left	99	10.0	Yes	59	5.9
Others	25	2.6	No	933	94.1
Signed a written labor contract with the employer			Having housing accumulation fund		
Yes	643	64.8	Yes	124	12.5
No	349	35.2	No	868	87.5
Number of jobs changed in 3 years			Number of houses moved in 3 years		
Never changed	629	63.4	Never changed	240	24.2
1–4	343	34.6	1–4	668	67.3
>4	20	2.0	>4	84	8.5
Having medical insurance			Having unemployment insurance		
Yes	479	48.3	Yes	186	18.8
No	513	51.7	No	806	81.2
Having endowment insurance			Having maternity insurance		
Yes	336	33.9	Yes	139	14.0
No	656	66.1	No	853	86.0
Having work-related injury insurance			Participated in community democratic management activities		
Yes	251	25.3	Yes	266	26.8
No	741	74.7	No	726	73.2
Participated in community democratic elections			Participated in party and League activities		
Yes	254	25.6	Yes	490	49.4
No	738	74.4	No	502	50.6

**Table 3:** The social circle composition of migrant women.

	Proportion of Urban Residents (%)				
	All	Majority	Half	Minority	None
Your friends	12.4	29.2	17.9	32.0	8.5
Your colleagues	10.8	30.6	19.7	30.7	8.2
Residents of your community	15.3	33.3	17.2	28.7	5.5
